# The dengue disquisition: A low-cost public housing conundrum in Klang Valley, Malaysia

**DOI:** 10.1371/journal.pone.0317349

**Published:** 2025-01-16

**Authors:** Nabila AbuBakar, Jerzy M. Behnke, Norhidayu Sahimin, Xiaoye Kang, Siti Nursyahirah Mohd Shahar, Yvonne Ai Lian Lim, Siti Nursheena Mohd Zain

**Affiliations:** 1 Institute of Biological Sciences, Faculty of Science, Universiti Malaya, Kuala Lumpur, Malaysia; 2 School of Life Sciences, University of Nottingham, University Park, Nottingham, United Kingdom; 3 Tropical Infectious Diseases Research and Education Centre (TIDREC), Universiti Malaya, Kuala Lumpur, Malaysia; 4 Department of Parasitology, Faculty of Medicine, Universiti Malaya, Kuala Lumpur, Malaysia; Universitas Syiah Kuala, INDONESIA

## Abstract

Dengue remains the most rapidly advancing vector-borne disease in the world, and while the disease burden is predominantly in low-to-middle-income countries, the association with poverty remains in question. Consequently, a study was undertaken to evaluate the prevalence of anti-dengue antibodies among individuals residing in the People’s Housing Program (PPR), a government-sponsored low-cost housing initiative targeting low-income earners. This type of public housing often faces challenges, including substandard housing facilities. Therefore this study took into consideration several social determinants of health, including the economic, environmental, and social conditions that contribute towards dengue transmission. The research was conducted over a period of 18 months across 14 PPRs in Klang Valley, Malaysia. Overall seroprevalence of anti-dengue immunoglobulin G (IgG) was 78.2% (CL: 72.5–83.1) among the 436 residents who participated in the study, while seroprevalence of anti-dengue IgM was 0.9% (CL: 0.2–3.2). Log-linear statistical models with the presence/absence of anti-dengue IgG and individual factors showed significant associations of anti-dengue IgG with age, income, location, and waste bin conditions, but ethnicity was just at the wrong side of the cut-off for significance. However, a multifactorial model, in which all relevant factors were taken into account, showed that location and ethnicity were the key risk factors. For anti-dengue IgM, the only significant association was with the presence of stagnant water bodies around the compounds. Findings from this study highlight an acute need for improvements in the environmental and societal health of those residing in PPRs in locations that are particularly at risk, and continuous community empowerment to ensure that the preventive measures taken to eradicate dengue are locally sustainable.

## Introduction

Dengue fever has long been endemic in Malaysia and Southeast Asia, with records of its existence in the region spanning over 120 years. According to the World Health Organization (WHO) [1], Southeast Asian countries, excluding the People’s Republic of Korea, account for half of the worldwide dengue burden. As a disease of poverty, dengue has long been the subject of extensive debate, with some studies finding a correlation between the prevalence of the infection and low socioeconomic status, income, and education [2], while others have failed to do so [3].

Dengue is no longer predominantly an urban disease, with comparable seroprevalence of dengue immunoglobulin G (IgG) in urban (61%– 92%) and rural (28%– 91%) settings [4, 5]. Nevertheless, it is essential to acknowledge that the primary dengue vector, *Aedes aegypti*, has thrived in areas of condensed, vertical living spaces [6]. Due to the adaptability of this species of mosquito to non-landed, high-rise buildings, and the increasing number of such facilities in urban cities, it is highly likely that these will create increasingly significant obstacles for controlling the vectors in the future. To some extent, this has been substantiated already by the findings of Lau et al. [7] and Ab Hamid et al. [8], who observed an abundance of *Aedes* larvae within residential high-rise buildings.

In the past five decades, the urbanisation rate in Malaysia has tripled from 28.4% in 1970 to 78% in 2022, with an urban population growth rate of 4% per annum [9, 10]. Klang Valley–comprising Wilayah Persekutuan (WP) Kuala Lumpur, WP Putrajaya, and Selangor, is the most densely populated metropolis in Malaysia, with urban populations of 100%, 100%, and 95.8%, respectively. While one might argue that urbanisation has the potential for a positive transformation, the number of urban poor is still relatively high. This may be attributed to the high migration of low-income groups into the big cities and the influx of foreign workers. Accelerated urban expansion over a short period may also increase urban diseconomies, including environmental deterioration, pollution, poor sanitation services, improper waste management, and population demands that outstrip service capacity [11].

Approximately 3 million of Malaysia’s 33.6 million population live in public or private low-cost housing schemes, with 1.76 million residing in Klang Valley. This means residents in low-cost housing schemes account for about a quarter of Klang Valley’s population [12, 13]. Although public housing remains the best option for low-income earners, offering low rents and allowing them to upgrade their living conditions and social mobility, following increased recent scrutiny, there are growing concerns about its effectiveness as it has created pockets of deprivation.

The present study targeted urban residents of the People’s Housing Project (Projek Perumahan Rakyat–PPR), a housing scheme developed by the Ministry of Housing and Local Government to house slum dwellers while also offering affordable low-cost housing to those within the lower-income bracket. PPRs in urban vicinities are typically of high-rise buildings with basic amenities, including clean water, latrines, and solid waste collection sites. A whisper in the realm of truth, yet galaxies apart in this narrative. The sad reality of today’s PPR is that they are often overcrowded and plagued with inadequate drainage, poor waste management, and unsustainable management of facilities, pushing the urban poor to live in echoes of decay, and allowing communicable diseases to thrive [14].

In 2023, 123,133 dengue cases were reported, including 100 deaths, representing an increase of over 70% compared to the same period in the previous year, when 57,031 cases and 56 deaths were reported [13], and the trend is expected to increase in the next year or so. Given the reality of the housing situation of the PPRs, this puts the urban poor at risk of infection. However, the reporting of disease incidences in Malaysia tends to reflect values for the mass community, as opposed to its marginalised group [15], thus creating a knowledge gap in disease prevalence. With this background, the current study was conducted to determine the anti-dengue seroprevalence in PPRs across Klang Valley and to identify associated risk factors in the targeted population. The results from this study will help highlight the sustainability of housing for the urban poor and offer insights into the current seroprevalence of this marginalised group.

## Methods

### Enrolment of participants and study population

This cross-sectional study was conducted from January 2022 to June 2023 in Klang Valley, Malaysia, an area with a high concentration of public housing.

The present study incorporated participants residing in public low-cost housing schemes developed by the Ministry of Housing and Local Government, commonly known as the People’s Housing Program (Program Perumahan Rakyat or PPR). A social wellness community program was conducted to recruit participants. Children under seven years old and those with contraindications for providing blood samples were excluded from this study. Apart from collecting samples and conducting interviews, physical screenings were performed, including measuring body mass index (BMI), blood pressure, and blood glucose levels. Social well-being was also promoted through educational talks on health issues and environmental cleanliness, and hygiene kits were provided for all participants.

### Estimation of sample size

The minimum sample size was estimated to be 192 participants based on a formula reported by Daniel [16] and based on earlier estimates of dengue seroprevalence (85.4%) in Malaysia’s urban settings [5], with a 95% confidence level and 5% margin of error. As recruitment was voluntary, 436 participants of all ages, sexes, and ethnicities from 14 different PPRs joined the study.

### Ethical consideration

All participants were fully informed about the nature of the study via the participants’ information sheets. Completed, written, and signed consent forms were obtained from each participant prior to screening. Consent for children was obtained from their parents or guardians before collecting data and providing blood samples. This study was approved by the Medical Ethics Committee of the Universiti Malaya Medical Centre (UMMC) (MRECID.NO: 20201210–9589) and the Universiti Malaya Research Ethics Committee (UMREC) (UM.TNC2/UMREC_1162).

### Data collection

Face-to-face interviews were conducted in each location, and structured questionnaires were presented to all participants after consent. The questionnaires comprised several sections, including socio-demographics, lifestyle habits and hygiene practices, history of illness and treatment-seeking behaviour, waste disposal practices, drinking water sources, and pet ownership. The participants’ knowledge, attitude, and practices regarding dengue were also assessed.

Approximately 3–6 mL of venous blood were drawn from each volunteer by medically trained personnel into a BD vacutainer (red top cap with non-anticoagulant) and kept in an icebox before being transported to the Parasitology Laboratory, Faculty of Science, Universiti Malaya, within 2–3 hours post collection. The blood samples were centrifuged at a fixed-angle-rotor centrifuge (Universal 320 Centrifuge, *Hettich* Laboratory, Germany) at 3,000 rpm for 10 minutes. Sera were aliquoted into tightly capped polystyrene tubes and stored at -20°C until use.

### Serological analysis

Seropositivity for Dengue Virus (DENV) infection was assessed by anti-dengue virus IgG and IgM antibodies using standard enzyme-linked immunosorbent assay (ELISA) commercial kits (EUROIMMUN Medizinische Labordiagnostika, Lubeck, Germany), following the manufacturer’s instructions. All reagents were maintained at room temperature for testing, and sera were allowed to thaw to the same temperature before testing commenced. For the IgG assay, a positive result was defined as a reading of 22 RU/ml, indicating a latent or past exposure to DenV, and a ratio reading of ≥ 1.1 showed a recent infection for the IgM assay.

### Data analysis

All data were analysed using the SPSS software version 23 (IBM, Armonk, NY). Summary data are provided for the prevalence of infection (percentage infected in relevant factor levels) plus 95% confidence limits (95% CL) calculated with bespoke software based on Rohlf and Sokal’s [17] statistical tables.

Statistical analyses were conducted using maximum likelihood techniques based on log-linear analysis of contingency tables. In the first phase, exploratory models were fitted with either the presence or absence of anti-dengue IgG or IgM and only one of the following factors in turn: sex (male or female), age class (<12, 13–18, 19–50, >50 years old), ethnicity (Malay, Chinese, Indian, and others). Other extrinsic factors taken into consideration were income level (US$ 1 = RM 4.68) [< RM1 000, RM1 000 –RM1 500, RM1 501 –RM2 000, RM2 001 –RM2 500, RM2 501 –RM3 000, >RM3 000, and not applicable], education attainment (no formal education, primary school, secondary school and university) and occupation status (employed and not employed). The lifestyle habits and hygiene practices recorded included sources of drinking water, drinking method, presence of stagnant water around the surrounding compound, occurrence of high-rise waste dumping, presence of stray animals, location and condition of waste collection facilities, and waste disposal practices. Other factors recorded included history of previous illness, treatment-seeking behaviour, and pet ownership.

After the initial round of exploratory analyses, multifactorial models were fitted incorporating the significant factors, and those with a *P* value of < 0.1, identified from the first round of analyses. Following stepwise backward selection, a minimum sufficient model (MSM) was generated, for which the likelihood ratio of Chi-square was not significant, indicating that the model was sufficient in explaining the data. The importance of each term in interactions involving the presence or absence of a positive IgG titre in the final model was assessed by the probability that its exclusion would alter the model significantly. These Chi-square values, with associated *P* values, are given in the text and tables, assessed by a likelihood ratio test between models with and without each term of interest.

Odds ratios (ORs) were calculated for anti-dengue IgG antibodies in bespoke software. One of each factor’s data subsets was selected as the reference cell to which all the remaining subsets were compared. We provide ORs with 95% confidence limits, *z* scores, and associated probabilities. Since so few subjects proved positive for anti-dengue IgM, it was not possible to calculate ORs for most data subsets.

## Results

### Sociodemographic characteristics

The present study spanned over a period of 18 months, commencing from January 2022 and concluding in June 2023, to achieve the required sample size. Volunteers (*n* = 436) hailing from 14 different PPRs participated (Table 1), with more than half females (63.1%). Most participants’ ages ranged between 18 and 50 years (62.8%), followed by those over 50 years accounting for 28.7%. More than half of these PPR residents were unemployed (59.6%), with secondary-level education (56.0%) as the highest education attainment, followed by primary-level education (31.4%).

**Table 1:** Study sites, parliamentary locations, and GPS coordinates.

No	Study sites	District	Coordinates
1	PHC	Shah Alam	3.03633, 101.54406
2	PDR	Bandar Tun Razak	3.07831, 101.71595
3	PSA	Lembah Pantai	3.09169, 101.67376
4	PPI	Lembah Pantai	3.09676, 101.67116
5	PCH	Lembah Pantai	3.10025, 101.67388
6	PSR	Lembah Pantai	3.10613, 101.67388
7	PKR	Lembah Pantai	3.10547, 101.66863
8	PPP	Lembah Pantai	3.10584, 101.67185
9	PKL	Lembah Pantai	3.10843, 101.67487
10	PHT	Bukit Bintang	3.13933, 101.70561
11	PSS	Bukit Bintang	3.1371, 101.70553
12	PLY	Bukit Bintang	3.1362, 101.70581
13	PBM	Batu	3.2058, 101.68044
14	PIB	Kepong	3.2345, 101.65442

### Seroprevalence of DENV infections and associated risk factors

**Fig 1:**
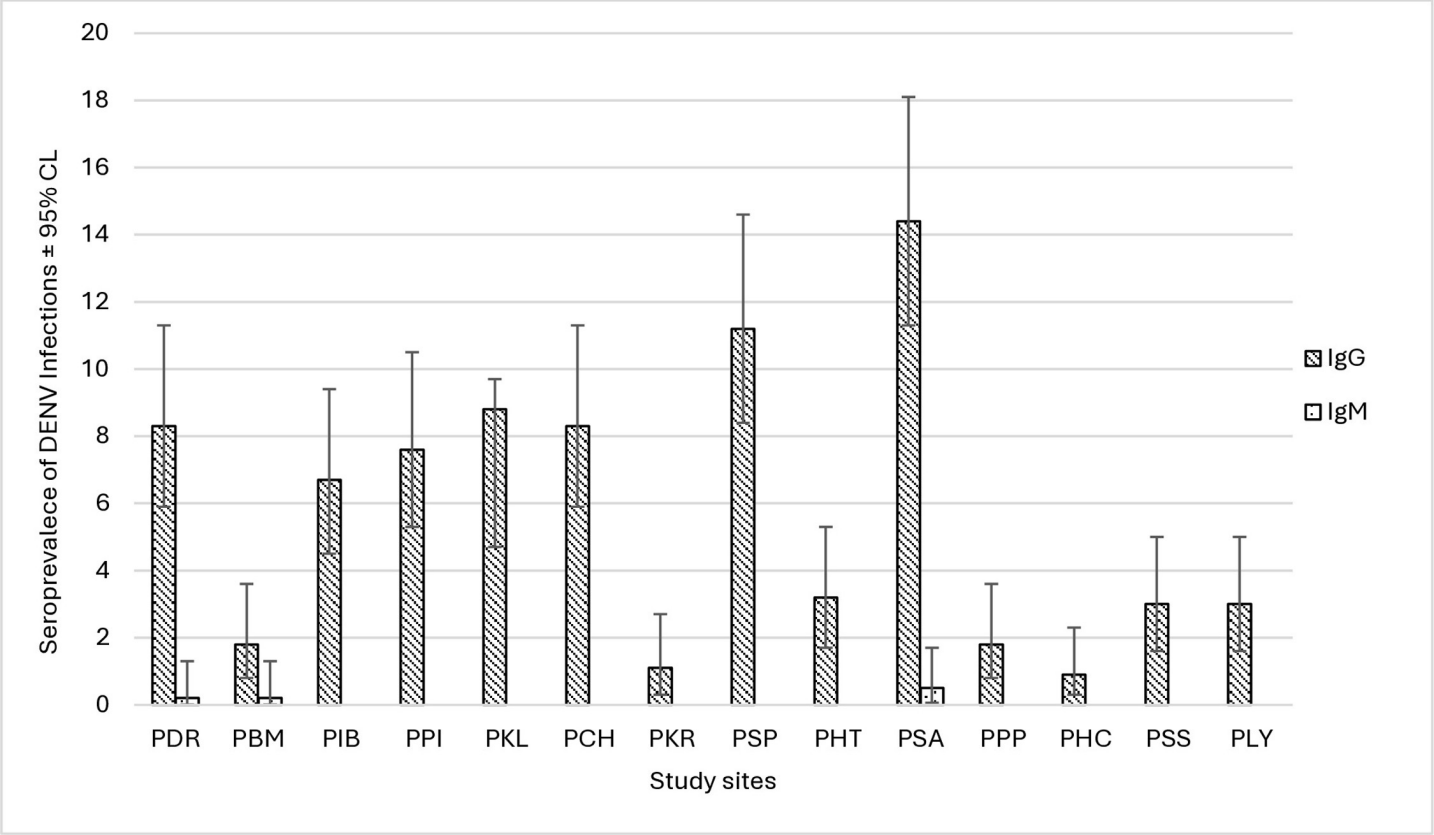
Breakdown of DENV seroprevalences according to study sites.

The seroprevalence of anti-dengue IgG and IgM was 78.2% (CL: 72.5–83.1) and 0.9% (CL: 0.2–3.2), respectively. The breakdown of seroprevalence of anti-dengue IgG and IgM according to locality is presented in Fig 1. Seroprevalence of DENV infections was then analysed statistically against sociodemographic, environmental and lifestyle habits and hygiene practice factors. In the initial phase of analysis, of the three intrinsic factors taken into consideration (sex, age, and ethnicity), only age (*χ*^2^_3_ = 20.944, *P*< 0.0001) was found to be significantly associated with seropositivity of anti-dengue IgG (Table 2). Compared with the youngest age class, seroprevalence was higher in adults >18 years of age, with ORs more than five times and significantly higher for the two older age classes compared with the younger (≤17 years old) subjects (Table 2).

**Table 2:** Potential risk factors on socio-demographics associated with IgG+ seropositivity of dengue infections in the urban poor population.

Factors	%	95% CL	*χ* ^2^	df	p-value	OR (95% CL)	z	P
**Sex**								
Male (n = 161)	78.3	70.2–84.7	<0.0001	1	0.985	1.005 (0.627, 1.610)	0.019	0.492
Female (n = 275)	78.2	73.7–82.1				1.000	-	-
Age*								
< 12 (n = 30)	46.7	29.8–65.2	20.944	3	**<0.0001**	1.167 (0.222, 6.136)	0.182	0.428
12–17 (n = 7)	42.9	12.9–77.5				1.000	-	-
18–50 (n = 274)	80.3	75.9–84.0				5.432 (1.181, 24.993)	2.173	0.015
> 50 (n = 125)	83.2	76.6–88.4				6.603 (1.376, 31.698)	2.358	0.009
**Ethnicity**								
Malay (n = 357)	77.3	72.1–81.8	6.746	3	0.080	1.603 (0.668, 3.851)	1.056	0.145
Chinese (n = 25)	68.0	48.0–83.9				1.000	-	-
Indian (n = 50)	88.0	74.7–94.0				3.451 (1.042, 11.428)	2.028	0.021
Others (n = 4)	100.0	47.3–100.0				-	-	-
**Education**								
No formal education (n = 14)	71.4	42.6–89.6	2.168	3	0.538	1.000	-	-
Primary school (n = 137)	75.2	67.6–81.5				1.212 (0.357, 4.115)	0.308	0.379
Secondary school (n = 244)	80.7	76.7–84.2				1.677 (0.504, 5.580)	0.842	0.200
University (n = 41)	75.6	59.3–87.2				1.240 (0.318, 4.837)	0.310	0.378
**Employment status**								
Yes (n = 176)	79 .0	70.7–85.7	0.102	1	0.749	1.079 (0.677, 1.718)	0.319	0.375
No (n = 260)	77.7	73.3–81.6				1.000	-	-
Income*								
<RM1000 (n = 101)	78.2	72.0–83.5	15.354	6	**0.018**	2.348 (1.084, 5.084)	2.165	0.015
RM1000-RM1500 (n = 76)	81.6	70.5–89.3				2.896 (1.247, 6.725)	2.473	0.007
RM1501 –RM2000 (n = 43)	60.5	43.6–75.6				1.000	-	-
RM2001 –RM2500 (n = 35)	82.9	68.7–91.8				0.542 (0.247, 1.188)	-1.531	0.063
RM2501 –RM3000 (n = 30)	93.3	78.7–98.8				9.154 (1.925, 43.535)	2.783	0.003
>RM3000 (n = 31)	87.1	74.6–94.1				4.413 (1.309, 14.876)	2.395	0.008
N/A (n = 120)	75.0	67.9–81.0				1.962 (0.938, 4.103)	1.790	0.037
Location*								
Petaling (n = 19)	21.1	7.5–44.6	47.057	5	**<0.0001**	1.000	**-**	**-**
Lembah Pantai (n = 267)	83.9	79.9–87.3				19.535 (6.184, 61.708)	5.065	<0.001
Bukit Bintang (n = 64)	62.5	51.4–72.5				6.250 (1.857, 21.033)	2.960	0.002
Bandar Tun Razak (n = 44)	81.8	65.2–91.6				16.875 (4.406, 64.634)	4.123	<0.001
Batu (n = 8)	100.0	63.5–100.0				-	-	-
Kepong (n = 34)	85.3	71.6–93.3				21.750 (5.076, 93.196)	4.148	<0.001

(*significant at 0.05)

The extrinsic factors considered included sociodemographic factors, notably education, employment status, household income, and study site locations. Household income was a significant factor for the seropositivity of anti-dengue IgG *(χ*^2^_6_ = 15.354, *P* = 0.018), with low earners (RM <1000–1500; US$ 1 = RM 4.68) and high earners (RM >3000) showing significantly different ORs from middle-level earners (RM1501-2500). Location was also a significant factor (*χ*^2^_5_ = 47.057, *P* < 0.0001), associated with seropositivity of anti-dengue IgG (Table 2). The data in Table 2 show that compared to the inhabitants of Petaling, ORs of anti-dengue IgG were significantly different in all the other four districts, with the least difference among those from Bukit Bintang, but similar higher values for the other three districts (ORs >16.9).

Of the lifestyle and environmental health factors taken into consideration (Table 3), only one factor was significantly linked with the IgG seropositivity: poor waste collection facilities (*χ*^2^_1_ = 4.509, *P* = 0.034), with an OR of 1.8 for those living in accommodation with poor waste bin conditions, almost twice that of subjects with good waste bin conditions.

**Table 3:** Potential risk factors on lifestyle and environmental health factors associated with IgG+ seropositivity of dengue infections in the urban poor population.

Factors	%	95% CL	*χ* ^2^	df	*p-value*	OR (95% CL)	z	P
**Drinking water sources**								
Pipe water (n = 362)	78.7	73.6–83.2	2.342	2	0.310	2.961 (0.776, 11.294)	1.589	0.056
Bottled water (n = 65)	78.5	67.9–86.3				2.914 (0.689, 12.321)	1.454	0.073
Others (n = 9)	55.6	25.1–83.1				1.000	-	-
**Drinking method**								
Boil (n = 167)	76.6	68.2–83.6	1.280	4	0.865	1.193 (0.360, 3.959)	0.289	0.386
Strain through cloth (n = 21)	85.7	64.6–96.0				2.182 (0.409, 11.641)	0.913	0.181
Filter (n = 233)	79.0	74.9–82.6				1.365 (0.417, 4.475)	0.514	0.303
Do nothing (n = 15)	73.3	46.6–90.3				1.000	-	-
**Presence of water bodies**								
Yes (n = 248)	77.8	73.6–81.6	0.051	1	0.821	1.000	-	-
No (n = 188)	78.7	70.0–85.6				1.054 (0.666, 1.671)	0.226	0.411
**High rise dumping**								
Yes (n = 330)	78.8	73.9–83.0	0.262	1	0.609	1.146 (0.681, 1.929)	0.515	0.303
No (n = 106)	76.4	69.8–82.1				1.000	-	-
**Stray animals at residential area**								
Yes (n = 205)	75.1	71.1–78.8	2.163	1	0.141	1.000	-	-
No (n = 231)	81.0	77.0–84.4				1.407 (0.892, 2.221)	1.468	0.071
**Pet ownership**								
Yes (n = 88)	77.3	64.5–86.6	0.057	1	0.812	1.000	-	-
No (n = 348)	78.4	73.4–82.8				1.071 (0.611, 1.875)	0.239	0.406
**Types of pets**								
Cats (n = 75)	76.0	64.3–85.0	1.715	3	0.634	1.000	-	-
Dogs (n = 3)	100.0	36.9–100.0				-	-	-
Others (n = 10)	80.0	44.7–96.3				1.263 (0.246, 6.496)	0.280	0.400
N/A (n = 348)	78.4	73.4–82.8				1.149 (0.638, 2.070)	0.464	0.321
**Live close to near waste disposal vicinity**								
Yes (n = 393)	78.9	73.5–83.5	0.993	1	0.319	1.446 (0.711, 2.938)	1.019	0.154
No (n = 43)	72.1	55.3–84.9				1.000	-	-
Condition of waste bins*								
Poor (n = 119)	84.9	78.7–89.6	4.509	1	**0.034**	1.800 (1.025, 3.162)	2.045	0.020
Good (n = 317)	75.7	70.8–80.1				1.000	-	-
**Waste disposal sites as breeding ground for pests**								
Yes (n = 362)	77.6	72.4–82.1	0.443	1	0.506	1.000	-	-
No (n = 74)	81.1	70.0–88.8				1.235 (0.657, 2.324)	0.655	0.256
**Overflow of rubbish in waste bins**								
Yes (n = 289)	79.9	75.4–83.8	1.464	1	0.226	1.340 (0.837, 2.145)	1.217	0.112
No (n = 147)	74.8	67.0–81.4				1.000	-	-
**Wastes around vicinity of residential area**								
Yes (n = 322)	78.0	73.1–82.2	0.049	1	0.824	1.000	-	-
No (n = 114)	78.9	72.4–84.5				1.061 (0.630, 1.787)	0.222	0.412
**Recent known infection of dengue**								
Yes (n = 7)	71.4	34.1–94.7	0.180	1	0.671	1.000	-	-
No (n = 429)	78.3	72.7–83.2				1.445 (0.276, 7.569)	0.436	0.331

(*significant at 0.05)

Only four subjects were positive for anti-dengue IgM. Analysis of anti-dengue IgM antibodies did not show any association with any of the three intrinsic factors (Table 4), and overall, only one significant factor was found: the presence of water bodies (i.e., puddles) in the vicinity of the PPRs (*χ*^2^_1_ = 4.542, *P* = 0.033). All four IgM-positive subjects lived near water bodies (Table 5).

**Table 4:** Potential risk factors on socio-demographics associated with IgM+ seropositivity of dengue infections in the urban poor population.

Factors	%	95% CL	*χ* ^2^	df	p-value
**Sex**					
Male (n = 161)	0.6	0.1–4.0	0.261	1	0.609
Female (n = 275)	1.1	0.4–2.8)			
**Age**					
< 12 (n = 30)	0.0	0.0–11.2	3.738	3	0.291
12–17 (n = 7)	0.0	0.0–37.7			
18–50 (n = 274)	1.5	0.6–3.3			
> 50 (n = 125)	0.0	0.0–2.3			
**Ethnicity**					
Malay (n = 357)	1.1	0.4–3.2	1.607	3	0.658
Chinese (n = 25)	0.0	0.0–13.4			
Indian (n = 50)	0.0	0.0–7.5			
Others (n = 4)	0.0	0.0–52.7			
**Education**					
No formal education (n = 14)	0.0	0.0–23.8	1.416	3	0.702
Primary school (n = 137)	1.5	0.3–5.0			
Secondary school (n = 244)	0.8	0.3–2.2			
University (n = 41)	0.0	0.0–9.5			
**Employment status**					
Yes (n = 176)	0.6	0.1–4.2	0.421	1	0.516
No (n = 260)	1.2	0.5–2.8			
**Income**					
<RM1000 (n = 101)	1.0	0.2–3.6	3.290	6	0.772
RM1000-RM1500 (n = 76)	1.3	0.1–7.8			
RM1501-RM2000 (n = 43)	0.0	0.0–9.9			
RM2001-RM2500 (n = 35)	0.0	0.0–8.4			
RM2501-RM3000 (n = 30)	3.3	0.2–17.7			
>RM3000 (n = 31)	0.0	0.0–7.7			
N/A (n = 120)	0.8	0.1–3.7			
**Location**					
Petaling (n = 19)	0.0	0.0–17.6	6.359	5	0.273
Lembah Pantai (n = 267)	0.7	0.3–2.2			
Bukit Bintang (n = 64)	0.0	0.0–4.8			
Bandar Tun Razak (n = 44)	2.3	0.1–14.3			
Batu (n = 8)	12.5	0.6–50.0			
Kepong (n = 34)	0.0	0.0–8.2			

(*significant at 0.05)

**Table 5:** Potential risk factors on lifestyle and environmental health factors associated with IgM+ seropositivity of dengue infections in the urban poor population.

Factors	%	95% CL	*χ* ^2^	df	p-value
**Drinking water sources**					
Pipe water (n = 362)	1.1	0.3–3.2	1.496	2	0.473
Bottled water (n = 65)	0.0	0.0–4.9			
Others (n = 9)	0.0	0.0–32.3			
**Drinking method**					
Boil (n = 167)	0.6	0.1–4.1	1.188	4	0.880
Strain through cloth (n = 21)	0.0	0.0–15.9			
Filter (n = 233)	1.3	0.6–2.9			
Do nothing (n = 15)	0.0	0.0–22.2			
Presence of water bodies*					
Yes (n = 248)	1.6	0.8–3.4	4.542	1	**0.033**
No (n = 188)	0.0	0.0–3.5			
**High rise dumping**					
Yes (n = 330)	0.9	0.3–2.8	0.001	1	0.974
No (n = 106)	0.9	0.2–3.6			
**Stray animals at residential area**					
Yes (n = 205)	1.0	0.4–2.3	0.014	1	0.905
No (n = 231)	0.9	0.3–2.3			
**Pet ownership**					
Yes (n = 88)	0.0	0.0–6.6	1.813	1	0.178
No (n = 348)	1.1	0.4–3.2			
**Types of pets**					
Cats (n = 75)	0.0	0.0–5.6	1.813	3	0.612
Dogs (n = 3)	0.0	0.0–63.2			
Others (n = 10)	0.0	0.0–29.1			
N/A (n = 348)	1.1	0.4–3.2			
**Live close to near waste disposal vicinity**					
Yes (n = 393)	1.0	0.3–3.2	0.835	1	0.361
No (n = 43)	0.0	0.0–9.9			
**Condition of waste bins**					
Poor (n = 119)	0.8	0.1–3.7	0.011	1	0.917
Good (n = 317)	0.9	0.3–2.8			
**Waste disposal sites as breeding ground for pests**					
Yes (n = 362)	1.1	0.3–3.2	1.496	1	0.221
No (n = 74)	0.0	0.0–5.5			
**Overflow of rubbish in waste bins**					
Yes (n = 289)	1.0	0.4–2.8	0.144	1	0.704
No (n = 147)	0.7	0.1–3.9			
**Wastes around vicinity of residential area**					
Yes (n = 322)	0.9	0.3–2.8	0.003	1	0.958
No (n = 114)	0.9	0.1–3.7			
**Recent known infection of dengue**					
Yes (n = 7)	0.0	0.0–37.7	0.130	1	0.718
No (n = 429)	0.9	0.2–3.2			

(*significant at 0.05)

Symptoms of infection (i.e. headaches, myalgia, and fever) indicated a strong association of IgG positivity with dengue (Table 6).

**Table 6:** Clinical symptoms associated with seropositivity of anti-dengue IgG+ and IgM+ in the urban poor population.

Factors	Level	IgG+		IgM+	
		% (95% CI)	p-value	% (95% CI)	p-value
Headache*	Yes (n = 239)	84.5 (79.3–88.9)	**<0.001**	0.4 (0.0001–2.3)	0.223
	No (n = 197)	70.6 (63.7–76.8)		1.5 (0.3–4.4)	
Jaundice	Yes (n = 4)	75.0 (19.4–99.4)	0.878	0.0 (0.0–60.2)	0.785
	No (n = 432)	78.2 (74.1–82.0)		0.9 (0.3–2.4)	
Myalgia*	Yes (n = 184)	87.5 (81.8–91.9)	**<0.001**	1.1 (0.1–3.9)	0.753
	No (n = 252)	71.4 (65.4–76.9)		0.8 (0.1–2.8)	
Chills	Yes (n = 61)	82.0 (70.0–90.6)	0.435	0.0 (0.0–5.9)	0.271
	No (n = 375)	77.6 (73.0–81.7)		1.1 (0.3–2.7)	
Diarrhoea	Yes (n = 106)	75.5 (66.2–83.3)	0.437	0.0 (0.0–3.4)	0.134
	No (n = 330)	79.1 (74.3–83.4)		1.2 (0.3–3.1)	
Abdominal discomfort	Yes (n = 130)	79.2 (71.2–85.8)	0.736	0.8 (0.0002–4.2)	0.830
	No (n = 306)	77.8 (72.7–82.3)		1.0 (0.2–2.8)	
Fever*	Yes (n = 312)	83.0 (78.4–87.0)	**<0.001**	1.3 (0.4–3.3)	0.101
	No (n = 124)	66.1 (57.1–74.4)		0.0 (0.0–2.9)	
Fatigue	Yes (n = 195)	80.5 (74.3–85.8)	0.293	1.0 (0.1–3.7)	0.832
	No (n = 241)	76.3 (70.5–81.6)		0.8 (0.1–3.0)	
Vomiting	Yes (n = 105)	79.0 (70.0–86.4)	0.811	1.0 (0.0002–5.2)	0.966
	No (n = 331)	77.9 (73.1–82.3)		0.9 (0.2–2.6)	
Rapid breathing	Yes (n = 54)	79.6 (66.5–89.4)	0.786	0.0 (0.0–6.7)	0.303
	No (n = 382)	78.0 (73.5–82.1)		1.0 (0.3–2.7)	
Rash	Yes (n = 19)	73.7 (48.8–90.9)	0.633	0.0 (0.0–17.7)	0.550
	No (n = 417)	78.4 (74.2–82.3)		1.0 (0.3–2.4)	

(*significant at 0.05)

### Multifactorial analysis of anti-dengue IgG and IgM

Multifactorial analyses of IgG seroprevalence comprised a model with host age, income, location, condition of waste collection facilities, and ethnicity. With all these factors considered, only location (*χ*^2^_5_ = 48.179, *P* < 0.001) and ethnicity (*χ*^2^_3_ = 7.868, *P* = 0.049) proved to retain significance. The failure of age (*χ*^2^_3_ = 2.605, *P* = 0.457), income (*χ*^2^_6_ = 9.822, *P* = 0.132), and condition of waste collection facilities (*χ*^2^_1_ = 2.493, *P* = 0.114) to retain significance in the multifactorial model emphasises the importance of controlling for possible confounding interactions between factors, that may give rise to misleading conclusions, when not controlled for in simpler statistical models.

For the analysis of IgM, only one factor (the presence of water bodies in the vicinity of PPRs) proved significant in the first round of analysis, and since none of the other initially tested factors showed *P* values less than 0.1, a multifactorial model was not relevant.

## Discussion

Dengue has been a public health issue in Malaysia since the 1970s and shows a cyclical pattern that peaks every four to five years, with the last peak occurring in 2019 [18]. Hence, it was anticipated that there would be a surge of dengue cases in the country by the end of 2023 or early 2024. This prediction proved accurate, as the number of cases in the fifth epidemiology week of 2024 was 18,427, compared to 11,127 cases reported in the same period last year [13]. However, dengue’s association with poverty remains in question. The findings of this study revealed a noteworthy seroprevalence of anti-dengue IgG of 78.2% amongst Malaysia’s urban poor communities living in low-cost public housing and identified location and ethnicity as the key risk factors. These results align with numerous studies conducted within other urban communities in Malaysia, indicating high seroprevalence rates of dengue [5, 19, 20].

An area is classified as a dengue hotspot when it experiences a sustained dengue outbreak lasting more than 30 days since the onset of the epidemic [21]. Upon examination of the Ministry of Health, Malaysia’s (MOH) archive for 2024 [13], it was found that out of 14 study locations over the past five years (2019–2023), nine were identified as dengue hotspots. Among these, three remained consistently active during the study period, with one study site–PBM, in the Batu district of Kuala Lumpur, particularly standing out in this respect. Interestingly, the current study also revealed a notably high presence of anti-dengue IgG antibodies among residents of PBM. However, a previous study conducted among PBM residents showed a paradoxical situation. While the residents exhibited a high level of dengue knowledge, their preventive practices were inadequate [22], potentially contributing to the persistent dengue outbreaks in the area. Notably, PBM has been listed as a dengue hotspot for three of the past five years.

Interestingly, the present study identified one PPR in the Petaling district–PHC, exhibiting the lowest DENV antibody prevalence (0.9%). This finding was unexpected, considering that the Petaling district has consistently reported the highest number of dengue cases in Selangor [18], and PHC has been listed as a dengue hotspot for four consecutive years. One plausible explanation could be that participants recruited for this study had recently acquired a good knowledge of dengue and implemented appropriate prevention practices.

**Fig 2:**
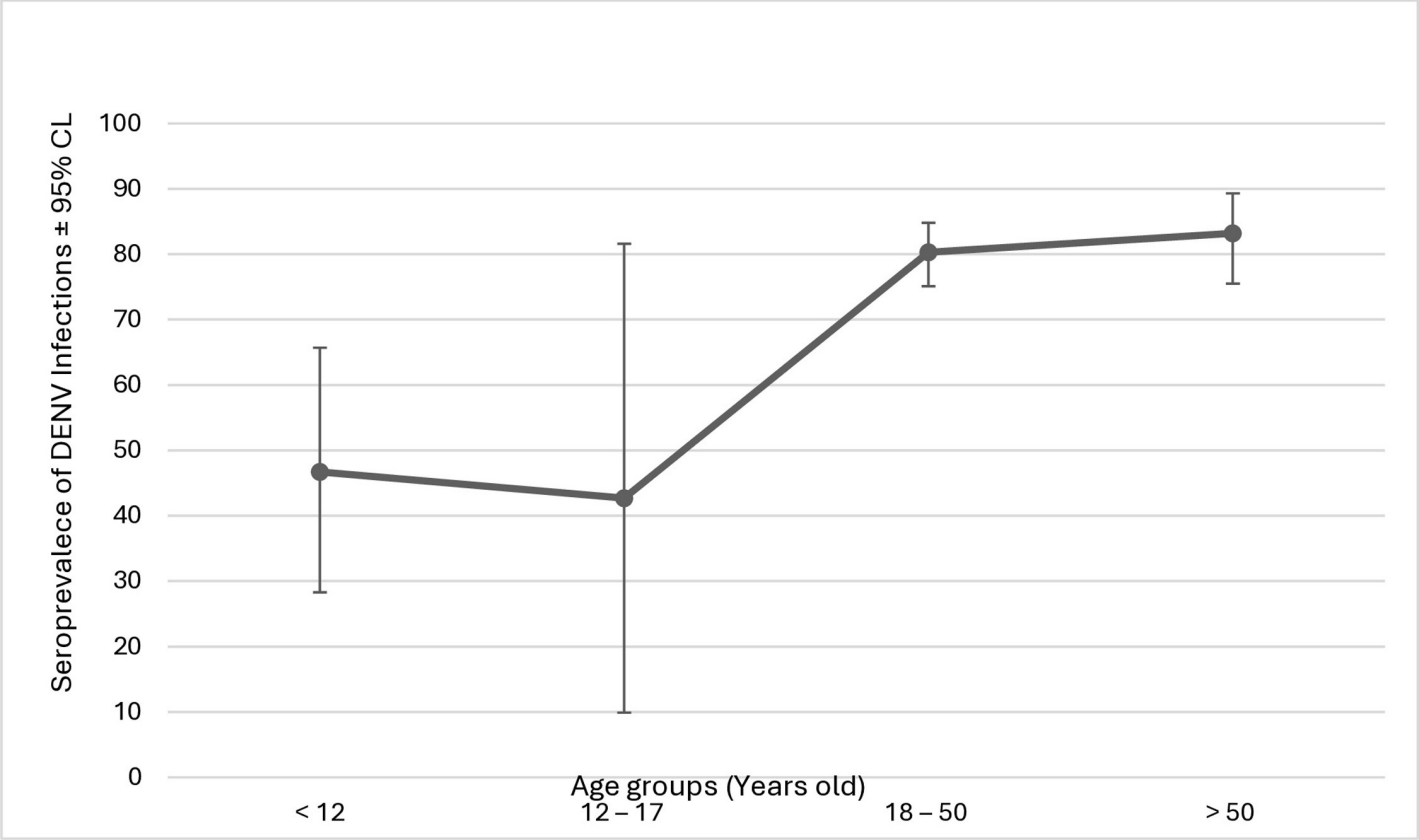
Anti-dengue IgG seroprevalence against age groups with 95% confidence interval.

Globally, dengue infections occur in all ages [23]. The present study initially identified age as a significant risk factor for anti-dengue IgG seroprevalence. Seropositivity was found to increase with age from young children to older age groups, peaking in the oldest age group (> 50 years of age; Fig 2). However, with other factors considered in the multifactorial model, age did not retain significance. Previous local and international studies have indicated that the longer a person stays in a dengue-endemic location, the greater the risk of exposure to the infection and the lifetime presence of anti-dengue antibodies [19, 24, 25]. This shift of seropositivity towards the older age groups in our study was likely attributable to the confounding influence of other factors. It might also have been due to underlying comorbidities that exacerbate infection, thus increasing the medical burden of dengue [26].

The present study also highlighted socioeconomic and environmental factors such as low-income, and poor housing conditions such as stagnant water and poor waste bin conditions as associated risk factors for anti-dengue IgG. Although this agrees with previous studies [27, 28] that pointed to an association of low-income and poor housing conditions with dengue outcomes [29], only location and ethnicity retained significance in our multifactorial model. Most PPRs in Klang Valley are plagued with indiscriminate waste disposal and poor management of waste collection facilities and drainage systems, which could very well contribute to dengue transmission in the community by providing breeding grounds for the vectors. Furthermore, the narrative of the association between poverty and dengue may instead mask the primary determinants of dengue’s spread, i.e., poor environmental health and local housing conditions, when, in fact, the presence of the *Ae*. *aegypti* vector is not exclusive to poor areas. The presence of stagnant water due to poor drainage systems within the vicinity of the PPR was also significantly associated with anti-dengue IgM, emphasising the importance of community involvement in maintaining environmental cleanliness to prevent mosquito breeding sites.

Mukhtar et al. [30] discovered that artificial, non-biodegradable water containers discarded in urban waste harboured the most *Aedes* larvae. Hence, aside from regular clearing of stagnant water, it is crucial to scrub clean water containers thoroughly to remove mosquito eggs, which tend to stick to the container walls.

The Malaysian government has also implemented communication for behavioural impact (COMBI), a community-oriented approach to tackling dengue. This approach mobilises all layers of community sectors and establishes a sense of shared responsibility to tackle dengue [31], similar to the Jumantik program done in the neighbouring country of Indonesia [32]. However, there is a need to re-evaluate the effectiveness of this program as cases of dengue continue to persist in Malaysia, especially in Klang Valley.

The cardinal signs reported with DENV infection include fever, myalgia, rash, headaches, vomiting, intense nausea, and bleeding [33–35]. This is confirmed in the current study, with headaches, myalgia, and fever as symptoms that were significantly present in volunteers who showed anti-dengue IgG positivity.

As we’ve discussed, dengue has been endemic in Malaysia for a long time. Hence, there is an acute need to understand the challenges that hinder the curbing of this infection. Moving forward, gauging the levels of knowledge, attitude, and practices against dengue amongst the community would be ideal for determining suitable community empowerment and interventions to reduce transmission. We recommend doing neutralisation assays to improve this study further and determine the DENV serotypes, as each has different virulence. By establishing the current viral profiles, we could better understand the cycles of the circulating predominant serotypes and better equip the health sector with the severity of possible infected cases.

To err is human; like any other study, this study has limitations as there were strengths. Firstly, the novelty of this research is that the study cohort is made up entirely of residents from government-sponsored low-cost high-rise housing projects, better known as PPRs. To the best of our knowledge, no study has yet been done on the prevalence of dengue exclusively amongst said cohort. Such study is essential as these communities are among the poorest in the country and are already subjected to other hardships, including poor housing facilities and abject living conditions. That being said, the screening was done cross-sectionally in selected PPRs across Klang Valley. Although the sample size might suffice to reflect the current situation in urban PPRs in Malaysia, it might not do justice to the sub-urban and rural PPRs.

## Conclusion

This study has identified location as the principal factor determining anti-dengue IgG positivity. Addressing dengue requires a comprehensive strategy, incorporating innovative technologies, overseeing and managing transmission, and maintaining community involvement as vital tools in the fight against the disease. This research marks a comprehensive investigation into the prevalence of dengue exclusively among residents in Malaysia’s high-strata, low-cost public housing and highlights a higher occurrence and increased risk of dengue due to suboptimal living conditions in some locations relative to others. Consequently, efforts for prevention and control should first focus on identifying the locations in which inhabitants are most at risk of infection and should be targeted thereafter at ensuring continuous and sustainable local community empowerment. This is crucial for translating knowledge into effective practices for eliminating dengue within these communities.

## Supporting information

S1 Raw data Seroprevalence of dengue raw data (436 participants). (XLSX)
